# Cerebral cavernous malformation remnants after surgery: a single-center series with long-term bleeding risk analysis

**DOI:** 10.1007/s10143-020-01436-7

**Published:** 2020-11-19

**Authors:** Marco M. Fontanella, Edoardo Agosti, Luca Zanin, Lodovico Terzi di Bergamo, Francesco Doglietto

**Affiliations:** 1 Division of Neurosurgery, Department of Medical and Surgical Specialties, Radiological Sciences and Public Health, University of Brescia, Brescia, Italy; 2 Division of Neurosurgery, Department of Biotechnology and Life Sciences, University of Insubria, Varese, Italy; 3 Institute of Oncology Research, Bellinzona, Switzerland

**Keywords:** Cerebral cavernous malformation, Bleeding risk analysis, Post-surgical bleeding

## Abstract

The aim of this work is to investigate the long-term bleeding risk of cerebral cavernous malformation (CCM) remnants. A review of clinical, radiological, operative, and post-operative data of a cerebral cavernous malformation (CCMs) prospective database was performed. Fisher’s exact test and Mann-Whitney *U*-test were used to assess differences between non-hemorrhagic and hemorrhagic CCM remnants for 14 variables. Recursive partitioning analysis was performed to assess the order of variables most associated with CCM remnant bleeding. Twenty-four patients out of 126 had a CCM post-surgical remnant. Of these, 7 had at least one post-operative hemorrhagic event. The mean follow-up was 80.7 months (range 12–144). CCM post-surgical remnant bleeding presented mostly with acute headache (50%) and focal neurological deficit (25%); in the remaining cases, the hemorrhage was asymptomatic. Retreatment was performed in two patients, with surgery and radiosurgery, respectively; no treatment was performed in the majority of cases. All patients ranked as non-II, according to Zabramski classification, did not show any post-surgical bleeding. The presence of a pre-operative perilesional hemosiderin ring was highly significant in predicting post-surgical bleeding (sensitivity = 0.94, specificity = 0.88) and incorrectly predicted bleeding in only two of the 24 patients. This study provides an evaluation of clinical and radiological factors influencing the bleeding risk of a CCM post-surgical remnant in a homogeneous population. Perilesional hemosiderin ring and Zabramski Type II appear to strongly condition the bleeding risk of a CCM post-surgical remnant.

## Introduction

Cerebral cavernous malformations (CCMs) are vascular lesions, histopathologically constituted by dilated vascular channels containing blood products at different stages and with a thin wall, formed by a single layer of endothelial cells and a delicate fibrous adventitia [1–3]. The tight junctions between the endothelial cells of these vascular caves are incomplete and dysfunctional, favoring bleeding of the lesion and the deposition of a perilesional hemosiderin ring [4, 5].

Complete surgical removal of a cerebral cavernous malformation (CCM) is mainly influenced by its size and location: lesions located in deep and/or eloquent areas might not be amenable to complete resection, leaving a lesion remnant in the surgical site [6–8]. CCM post-surgical remnants may be associated with various complications, including bleeding [9] and seizure [10].

Data on factors that can influence the bleeding risk of CCM remnants are limited [11, 12], and most of the studies focus on brainstem CCM remnants [13–16]. In this single-center study, based on a prospective registry of consecutive patients operated in our center, anamnestic, clinical and radiological factors were analyzed, and their possible association with the bleeding risk of CCM remnants was investigated.

## Materials and methods

Patients who were diagnosed with a CCM post-surgical remnant on post-operative magnetic resonance imaging (MRI) were selected from a prospective database, created in 2008 at the University of Brescia.

### Surgery

Surgical treatment was proposed according to the Hernesniemi grading system [17]. Radiological diagnosis was confirmed by histopathological examination. An early postoperative computer tomography (CT) scan and a 3-month MRI were performed for each patient; follow-up MRIs were usually performed every year for the first 5 years, especially if a remnant was suspected. A CCM remnant was diagnosed when a blood-containing nodule was recognized close to the surgical cavity, according to Chen B. et al. [18].

### Data collection and analysis

Anamnestic, clinical, and radiological factors were retrieved from the prospectively collected database. Anamnestic and clinical data included bleeding clinical onset, patient comorbidities, pre- and post-operative drugs (i.e., antiepileptic, antiplatelet, anticoagulant, antihypertensive, hypoglycemic, cholesterol-lowering), smoke or drug addiction, CCM familiarity, and postoperative radiation therapy. Radiological data included CCM localization, pre-operative maximum diameter, presence, or absence of a perilesional pre-operative hemosiderin ring. Zabramski classification was used to rank each CCM [19]. Three-tied multi-parameter MRI-based grading system for eloquent CCM was also considered for the analysis [20].

### Statistical analysis

Patients with CCM post-surgical remnant were divided into two groups based on whether or not the remnant bled. Differences between these two groups and baseline clinical variables were assessed using Fisher’s exact test, for discrete variables, and Mann-Whitney *U*-test, for continuous variables. A recursive partitioning analysis was used to identify the order of variables most associated with post-surgery bleeding. For the assessment of binary classification, sensitivity, specificity, positive predictive value (PPV), and negative predictive value (NPV) were calculated. All statistical tests were two-sided. Statistical significance was defined as a *p* value < 0.05. The analysis was performed with R statistical software v3.4.1 (http://www.r-project.org).

## Results

Out of 126 patients, who were treated at the University of Brescia from 2008 to 2018, 24 had a CCM post-surgical remnant (Table 1). The mean follow-up period was 80.7 months (range 12–144 months). Of the 24 patients, 16 did not show any bleeding, while 7 had at least one CCM post-surgical remnant bleeding. Most patients had a single bleeding episode, and only one patient presented two distinct hemorrhages. CCM post-surgical remnant bleeding was observed at a mean of 21.5 months (range 5–48 months) after surgery.

**Table 1:** Baseline characteristics of the 23 patients’ cohort with CCM remnants. Pre-operative drugs included antihypertensive, hypoglycemic, cholesterol-lowering, and gastroprotectors; post-operative drugs included antiepileptic, antihypertensive, neurotropic, anti-inflammatory, and analgesics

Variable		*N*/mean	%/95%CI
Gender	Male	12	50.0%
Localization	Frontal	5	20.8%
	Parietal	5	20.8%
	Temporal	6	25.0%
	Limbic	5	20.8%
	Insular	2	8.4%
	Cerebellar	1	4.2%
ICH clinical onset	Yes	14	58.4%
Zabramski type II	Yes	10	41.6%
Hemosiderin ring	Yes	7	29.1%
Comorbidities	Yes	10	41.6%
Medications pre	Yes	10	41.6%
Medications post	Yes	11	45.8%
Smoke	Yes	5	21.7%
Drugs	Yes	1	4.3%
CCM familiarity	Yes	5	20.8%
Post-operative RT	Yes	1	4.2%
Age		41.7	4–78
Pre-operative size		22.3	5.6–52

There was no sex preponderance; the mean age was 41.7 years (range 4–78). Fourteen patients presented with intracerebral hemorrhage (ICH = 56.3%), and 5 patients (21.7%) had CCM familiarity. CCM localization is reported in Table 1: deep location (58%) and dominant hemisphere locations (64%) were the most common. The mean pre-operative maximum diameter of the CCMs was 21.1 mm (range 5.6-52 mm). Ten patients were classified as type II according to Zabramski’s classification system (43.5%). In 8 patients, a perilesional hemosiderin ring was evident.

Bleeding of the CCM remnant was symptomatic in 75% of cases, occurring mostly with an acute headache (50%) and in a lower percentage of cases (25%) with the appearance of focal neurological deficit; in these cases, the bleeding was extracapsular. In the remaining cases of CCM remnant, bleeding was diagnosed during the radiological follow-up and was intracapsular (25%).

Two patients underwent treatment after the CCM remnant bleeding. A 25-year-old female patient, with a left temporal CCM remnant, presented with hemorrhage 24 months after the first treatment; she has been operated again with the resolution of bleeding and partial excision with no further bleeding (108 months of follow-up after second surgery). A 30-year-old male patient with a left cerebellar peduncle CCM remnant, who bled 12 months after surgery, underwent radiosurgical treatment, with the resolution of bleeding and ablation of part of the remnant (follow-up after radiosurgery, 136 months).

**Fig. 1:**
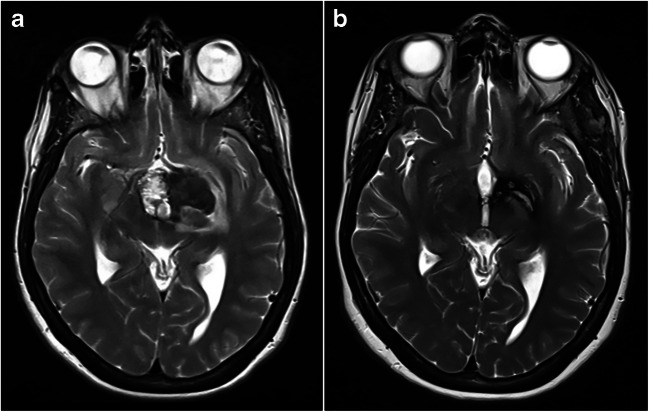
Axial T2WI head-MRI scan**. a** Pre-operative head-MRI scan. CCM located between the hypothalamus, the third ventricle, and the perimesencephalic cisterns with recent bleeding causing mass effect and third ventricle displacement of 7 mm. **b** Post-operative head MRI scan. Post-surgical malacic area at the level of the left lenticular nucleus; in the interpeduncular cistern nodule with an axial diameter of 10 × 4 mm compatible with residual cavernoma

The remaining five patients with bleeding CCM remnants did not undergo any further procedure. Two reported new neurological deficits (i.e., ideomotor slowing, space-time disorientation, hemianopsia, hyposthenia), while the other three patients had no relevant clinical consequences. An example of our CCM remnant series is shown in Fig. 1.

Furthermore, we have applied the three-tied multi-parameter MRI-based grading system for eloquent CCM [20]. Eighteen CCMs were classified as type 2, 1 CCM was classified as type 1, and 4 CCMs were classified as type 3. We were not able to prove a statistically valid correlation (*p* < 0.05) between the risk of bleeding and the belonging to one of three Dammann types.

### Statistical analysis

Out of the 14 assessed variables, the Zabramski score and the presence of a hemosiderin ring were significantly different between the non-bleeding and bleeding groups. Patients with post-surgery bleeding were all Zabramski type II, and 88% showed the presence of a hemosiderin ring (Table 2). The two variables were highly correlated, as suggested by Fisher’s exact test (*p* < 0.001) and the mean square contingency coefficient (Φ = 0.77).

**Table 2:** Baseline differences between CCM remnant bleeding patients and non-bleeding patients

Bleeding event							
		No (*N* = 16)		Yes (*N* = 7)			
Variable		N/mean	%/SD	N/mean	%/SD	Missing	*P* value
Gender	Male	7	43.8%	5	71.4%		0.684
Localization	Frontal	4	25.0%	1	14.3%		0.668
	Parietal	4	25.0%	1	14.3%		
	Temporal	2	12.5%	3	42.9%		
	Limbic	4	25.0%	1	14.3%		
	Insular	2	12.5%	0	0.0%		
	Cerebellar	0	0.0%	1	28.6%		
ICH clinical onset	Yes	8	50.0%	6	85.7%		0.286
Zabramski type II	Yes	3	18.8%	7	100.0%		< 0.001
Hemosiderin ring	Yes	1	6.3%	6	85.7%		< 0.001
Comorbidities	Yes	9	56.3%	1	14.3%		0.079
Medications pre	Yes	8	50.0%	2	28.6%		0.388
Medications post	Yes	8	53.3%	3	42.9%	1	0.667
Smoke	Yes	4	25.0%	1	14.3%		0.631
Drugs	Yes	1	6.3%	0	0.0%		1
CCM familiarity	Yes	4	25.0%	1	14.3%		0.631
RT post	Yes	0	0.0%	1	14.3%	1	0.348
Age		43.3	16.8	38.5	15.8		0.624
Size pre-op (mm)		21.1	12.6	21.6	10.1		0.783

Other clinical and anamnestic patient factors (i.e., age, familiarity, pre- and postoperative medications, postoperative radiotherapy (RT), cardiovascular and general comorbidities, voluptuary habits and other lesion parameters, i.e., initial clinical presentation with ICH and pre-operative size) were not significantly associate to the bleeding event (Table 2).

**Fig. 2:**
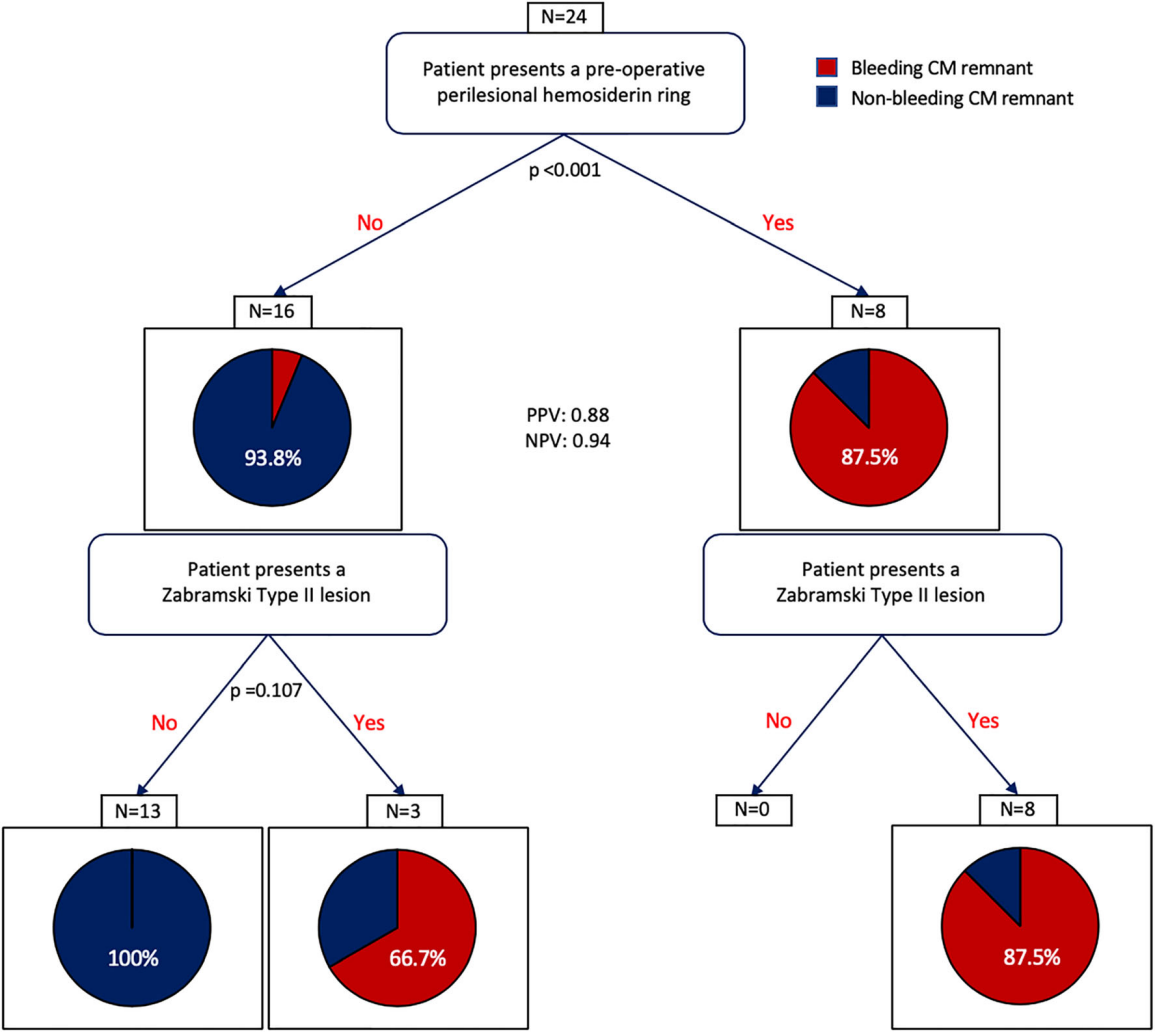
Recursive partitioning analysis identifies the presence of a hemosiderin ring as the most important variable in predicting post-surgical bleeding risk

A recursive partitioning analysis was carried out in order to identify the most significant variable in predicting post-surgery bleeding. The presence of a hemosiderin ring was selected as the most predictive variable (Fig. 2). However, 100% of the non-Zabramski type II patients did not show bleeding after surgery.

While perfect separation was not observed in any of the two variables considered with regard to post-surgery bleeding, the presence of a hemosiderin ring correctly predicted the outcome of 93.8% of patients, in contrast to Zabramski’s classification which misidentified 3 patients. The presence of a hemosiderin ring yielded a sensitivity of 0.94, a specificity of 0.88, a PPV of 0.88, and an NPV of 0.94, in contrast to Zabramski type II that showed a sensitivity of 0.81 and a specificity of 1, a PPV of 0.72, and an NPV of 1.

A model including both variables increases neither sensitivity nor specificity. No observable characteristics were identified in these two patients that could explain the missing correlation between the hemosiderin ring and bleeding.

## Discussion

In this consecutive cohort of patients with CCM post-surgical remnants, the presence of hemosiderin ring and the Zabramski Type II proved the strongest predictive variables for the occurrence of post-surgical bleeding. The presence of a pre-operative perilesional hemosiderin ring is the most predictive factor of future bleeding of a CCM post-surgical remnant (Fig. 1).

The bleeding risk of CCM post-surgical remnants remains a point of open debate among the authors, with estimates reporting 40% of both cerebral and brainstem CCM remnants with at least one bleeding episode (follow-up range of 6–32 months) [9, 12].

Currently, shared guidelines on the treatment of CCM have not yet been defined, and a common opinion is still lacking, especially regarding the treatment of supratentorial CCM without cavernoma-related epilepsy [21]. In particular, in this case, the therapeutic options include open surgery and/or stereotactic radiosurgery (SRS) [21]. In the case of CCMs related to medically refractory seizures, CCM surgical resection can be a resolving therapeutic option [22]. Indeed, it has been shown that total lesionectomy results in a 70 to 90% postoperative seizure control in patients with sporadic seizures or those with seizure duration less than 1 year [23]. Conversely, there is a lower chance of seizure control after surgery in cases with longer pre-operative duration of seizures [24]. In line with these data, some authors argue for performing early surgery in patients who do not respond to single-drug therapy, even if they do not satisfy criteria for CCM medically refractory epilepsy. SRS has been proposed as an alternative treatment for symptomatic CCM in eloquent areas [25]. A meta-analysis identified 4 out of 5 studies revealing statistically significant decline in the yearly hemorrhage rate 2 years after SRS of brainstem CCM [26]. The mortality rate was 5.61%, and 11.8% developed new focal neurological deficits. An active debate as to whether the effects of SRS merely reflect the CCM natural history is ongoing.

Several factors have been previously associated with CCM bleeding risk, such as prior hemorrhage, [3, 5, 27, 28], multiplicity of CCMs [27], male gender [28], deep and subcortical location [29], and pregnancy [5]. Furthermore, the correlation between perilesional hemosiderin ring and CCM bleeding is not new in the literature [12, 30] and might be intuitive, considering that hemosiderin rings are the expression of the previous micro- and macrohemorrhages deriving from the structural instability of CCM vascular caves. However, no study describes the association between perilesional pre-operative hemosiderin ring and bleeding of CCM post-surgical remnants. A probable reason for the lack of this type of study can be found in the reduced number of CCM post-surgical remnant cases available in prospective registries. The relatively high percentage of this study might be related to the stringent definition of radiological remnant used to evaluate post-operative images [18] and to the location of the operated CCMs, which are mostly located in eloquent and deep areas.

The correlation between perilesional pre-operative hemosiderin ring and bleeding risk of CCM post-surgical remnant most likely has the same histopathological explanation (i.e., architectural instability of caves walls). The greater the instability of the CM vascular caves, the greater the perilesional hemosiderin ring, the higher the risk of bleeding.

According to the Zabramski neuroradiological grading of CCM [19], type II are lesions with typical “popcorn” appearance, with both hemorrhages and thromboses in different stages. No previous studies documented a statistically significant association between Zabramski type II pre-operative grade and higher bleeding risk of CCM post-surgical remnants. Rather, Zabramski type II lesions have been previously correlated to the clinical presentation with seizures of CCMs [29].

In this study, we underline the clinical and surgical relevance of perilesional hemosiderin ring and Zabramski type II CCMs. These variables statistically strongly condition the bleeding risk of a possible post-operative remnant, so much that the presence of these two factors should direct the surgeon toward complete removal of the lesion. However, complete surgical removal of a Zabramski type II lesion with hemosiderin ring is not an easy task, certainly from a histopathological point of view, having a high tendency to bleed, to which is added a possible localization in eloquent areas with the impossibility of complete removal in order to guarantee an adequate postoperative outcome [31]. In the context of CCM with these two pre-operative neurovascular variables, the correct path is to find the right balance between obtaining a complete removal and avoiding surgical damage to the healthy parenchyma, avoiding where practicable to leave remnants of the lesion. We believe the combination of pre-operative perilesional hemosiderin ring and Zabramski type II lesion are two possible predictors of bleeding risk of the CCM post-surgical remnant.

### Limitations of the study

The main limitation of this study is represented by the small sample. We cannot exclude that the low number of patients may have hindered the identification of other risk factors for post-operative delayed bleeding in CCM remnants. The single-center analysis may also have limited the variability of the sample.

## Conclusions

This study offers an analytical evaluation of clinical factors influencing the bleeding risk of a CCM post-surgical remnant in a homogeneous population of patients. The presence of a pre-operative hemosiderin ring and Zabramski type II lesion are significantly associated with the bleeding risk of the post-surgical remnant. Pre-operative perilesional hemosiderin ring has shown the greatest correlation. With the identification of these two statistically significant risk factors, we aim to give the surgeon an extra weapon in the therapeutic decision. Having to decide whether to completely remove a CCM risking neurological damage, or leave a residue knowing that there is a risk of bleeding, according to our data, the presence of hemosiderin ring and Zabramski type II are risk factors that could push toward total removal. These data will need to be confirmed by larger, multicenter series.
